# Unveiling the microbiota of sauce-flavor *Daqu* and its relationships with flavors and color during maturation

**DOI:** 10.3389/fmicb.2024.1345772

**Published:** 2024-01-24

**Authors:** Weiwei Dong, Xiang Yu, Luyao Wang, Menglin Zou, Jiyuan Ma, Jun Liu, Yanli Feng, Shumiao Zhao, Qiang Yang, Yuanliang Hu, Shenxi Chen

**Affiliations:** ^1^Hubei Key Laboratory of Edible Wild Plants Conservation and Utilization, College of Life Sciences, Hubei Normal University, Huangshi, China; ^2^Hubei Key Laboratory of Quality and Safety of Traditional Chinese Medicine Health Food, Jing Brand Co., Ltd., Daye, China; ^3^State Key Laboratory of Agricultural Microbiology and College of Life Science and Technology, Huazhong Agricultural University, Wuhan, China

**Keywords:** *Daqu* maturation, microbial community, *Kroppenstedtia*, color formation, pyrazines

## Abstract

This study investigated the microbial community in three-color sauce-flavor *Daqu* (black, yellow, and white) throughout their maturation processes, together with their physicochemical factors, culturable microbes, flavor components, and fermenting vitalities. Results from high-throughput sequencing revealed distinct microbial diversity, with more pronounced variations in bacterial community than in fungal community. Firmicutes and Ascomycota emerged as the most dominant bacterial and fungal phyla, respectively, during maturation. Genus-level analysis identified *Kroppenstedia*, *Virgibacillus*, and *Bacillus* as dominant bacteria in black *Daqu*, yellow *Daqu*, and white *Daqu*, severally, while *Thermoascus* was shared as the core dominant fungi for these *Daqu*. Physicochemical factors, particularly acidity, were found to exert a significant impact on microbial community. *Kroppenstedtia* was the key bacteria influencing the color formation of these *Daqu*. Furthermore, correlations between dominant microbes and flavor compounds highlighted their role in *Daqu* quality. Molds (*Aspergillus*, *Rhizomucor*, and *Rhizopus*), excepting *Bacillus*, played a crucial role in the formation of pyrazine compounds. Consequently, this study offers innovative insights into the microbial perspectives on color and pyrazine formation, establishing a groundwork for future mechanized *Daqu* production and quality control of sauce-flavor baijiu.

## Introduction

Baijiu, one of the six globally recognized distilled spirits, has gained a lot of attention owing to its profound historical roots and cultural significance in China (Yan et al., 2021). Recent data from National Bureau of Statistics revealed the impressive scale of this industry, with the year 2022 witnessing a baijiu production of 6.71 billion liters, considerably contributing to national economy (National Statistics Bureau, 2023). Remarkably, sauce-flavor baijiu, is the leader among the three major categories of baijiu flavor, including light-flavor, strong-flavor, and sauce-flavor. The increasing interest among national consumers in sauce-flavor baijiu is largely driven by its exceptional quality and pleasurable drinking experience. The intricate and traditional craftsmanship involved in brewing sauce-flavor baijiu plays a pivotal role in producing high-quality baijiu. The intricate brewing processes encompass several essential stages, consisting of *Daqu* production, solid-state fermentation, distillation, storage and aging, and blending (Jin et al., 2017; Niu et al., 2022; Yang et al., 2023). As the adage goes, “*Daqu* is the backbone of baijiu brewing.” emphasizing the crucial role of *Daqu*—a fermented agent initiating the solid-state fermentation of baijiu by introducing a diverse array of microbes, enzymes, and flavor precursors, ultimately determining the final quality of base liquor (De Melo et al., 2019; Xia et al., 2022; Li H. et al., 2023; Yang et al., 2024).

**Figure 1:**
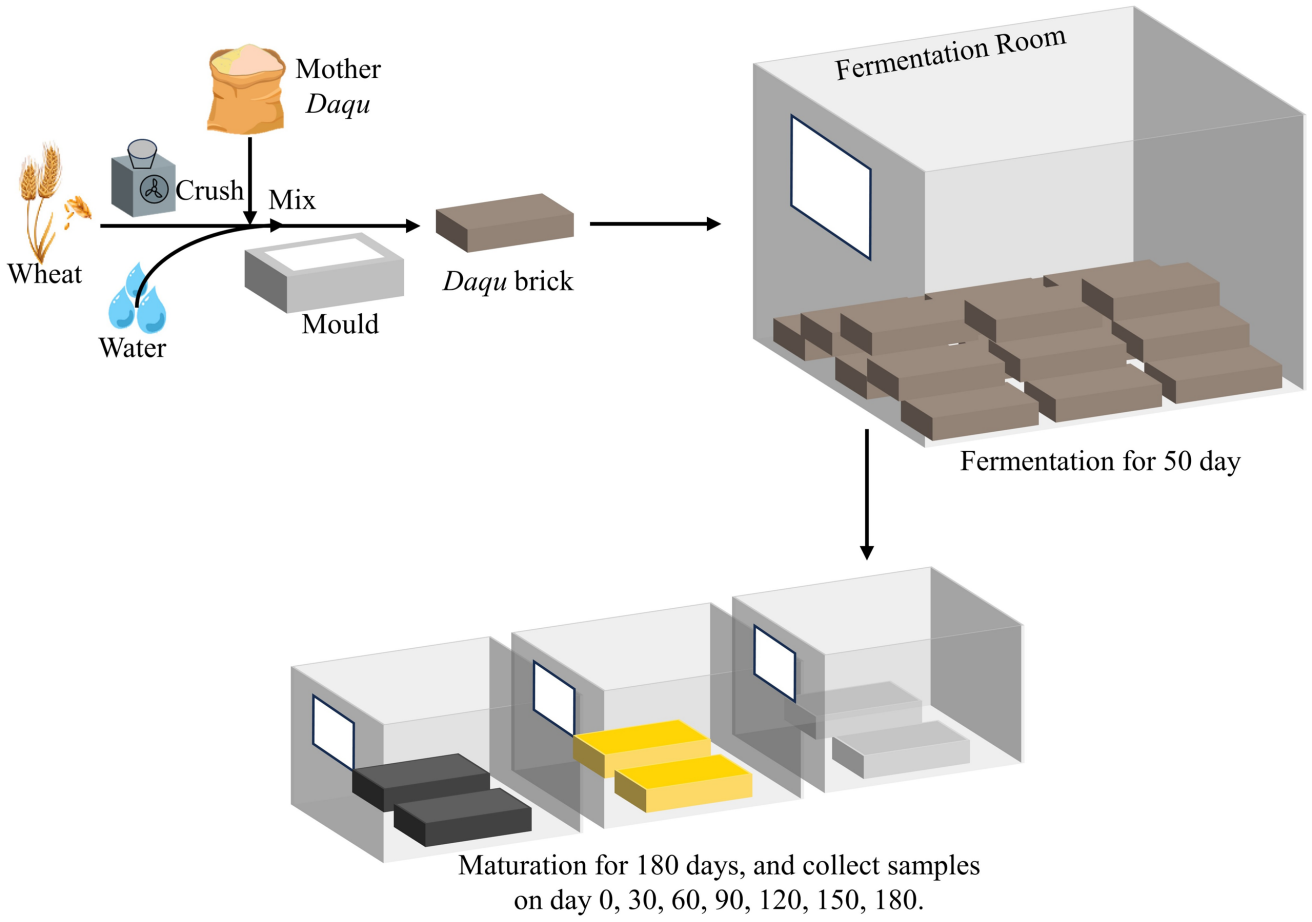
The schematic diagram of *Daqu* production.

In the context of sauce-flavor baijiu brewing, the *Daqu* employed is known as high-temperature *Daqu*, signifying its production under elevated temperature conditions (above 60°C) (Deng et al., 2020; Sakandar et al., 2020; Pan et al., 2023). The essential processes involved in the production of this Daqu are illustrated in Figure 1. Wheat is crushed, mixed with 34–40% water and 5–8% mother-*Daqu* powder, and then molded into *Daqu* bricks. These bricks undergo a 50-day high-temperature fermentation in a dedicated chamber, followed by a 6-month maturation period in a storage room (Niu et al., 2022). After maturation, the *Daqu* is crushed and used in the production of sauce-flavor baijiu, highlighting the critical role of fermentation and maturation in determining *Daqu* quality. Notably, the production of *Daqu* involves spontaneous processes with minimal human intervention, leading to the natural formation of three distinct *Daqu* color: black, yellow, and white (Cai et al., 2021; Shi W. et al., 2022). Previous studies have delved into the difference in microbial community of these three kinds of *Daqu* during fermentation process. Zhu’s study uncovered specific insight in the microbial community succession during *Daqu* fermentation (Zhu C. et al., 2022). High temperature emerged as the most significant factor driving core functional community of *Daqu*, correlating with flavor formation throughout fermentation (Zhu Q. et al., 2022; Wu et al., 2023). Others studies compared the differences in microbial community and flavor between artificial and mechanical *Daqu* during fermentation (Huang et al., 2023). Deng’s research shed light on the discrepancies in microbial composition among colored *Daqu* (Deng et al., 2020). In addition, several studies unveiled the potential factors contributing to color formation in the three types of *Daqu*, highlighting the significant roles played by amino acid metabolism and the Maillard reaction (Zhang et al., 2022; Zhu Q. et al., 2023).

Despite the big differences in the properties of the three-color *Daqu*, they are used in combination with specifical proportions to ensure the stable brewing and producing of sauce-flavor baijiu. These *Daqu* types undergo spontaneous fermentation, presenting challenges in controlling color formation and hindering the targeted production of specific *Daqu* varieties, impacting future quality control efforts. While previous researches have primarily focused on the microbiota of these *Daqu* during the fermentation process and proposed potential reasons for color formation, insufficient attention has been given to the maturation process, specifically regarding microbial composition, flavor components, and vitalities. Additionally, the microbes associated with color formation remain unclear. This study is dedicated to exploring the maturation process of these three *Daqu* types, investigating their physicochemical properties, cultivable microbes, fermenting vitalities, flavor substances, and corresponding microbial community. Hence, a systematically comparing analysis was carried out to reveal the microbiota differences among these colored *Daqu*, uncovering the core microbes related to color formation and laying a foundation for targeted production of specific *Daqu* and enhanced quality control in the future.

## Materials and methods

### Daqu maturation and sampling

The *Daqu* bricks were fermented in the *Qu*-producing shop of Jing Brand Maotai Town Liquor Co., Ltd., Renhuai, Guizhou province, China. Following fermentation, *Daqu* was categorized by color (black, yellow, and white) and moved to the storage room for maturation with a period of 180 days. Samples were collected at intervals of 0, 30, 60, 120, 150, and 180 days from each color of *Daqu*, resulting in 54 samples (three replicates per color) for subsequent analysis. Once the samples were gathered, the brick from each kind of *Daqu* was crushed to powder for the immediate assessment of physicochemical properties, cultivable microbes, and fermenting vitalities. The powder was stored at −80°C for subsequent flavor compounds analysis and amplicon sequencing.

### Physicochemical properties analysis

The analysis of moisture, acidity, and reducing sugar content in three kinds of *Daqu* were conducted based on our previous study with minor revisions (Dong et al., 2022). The amino acid nitrogen content of *Daqu* was detected following the methodology outlined in Huang’s study (Huang et al., 2021). Specifically, moisture content was measured by taking 4 g of *Daqu* powder in an oven for desiccation (overnight) until a constant weight, and calculating the ratio of weight loss. Before the acidity assessing, 10 g of *Daqu* powder was added into 200 mL of ddH_2_O with 30 min of static for acids extraction. Subsequently, the supernatant was used for acid–base titration to determine the acidity. The content of reducing sugar in *Daqu* was evaluated via Fehling’s test in reliance on the standard reducing sugar solution. As for the amino acid nitrogen, it was evaluated based on the amphoteric behavior of amino acids. Firstly, the amino acid nitrogen was extracted form *Daqu* by mixing 20 g of samples into 60 mL of ddH_2_O together with vibration at 120 rpm for 30 min, and the filtrate was collected and used for detecting. Subsequently, the pH value of filtrate was adjusted to 8.2 with 50 mM NaOH, together with 10 mL of methanal being added to fix the amino base. Finally, the titration with standard NaOH solution was performed to determine the end-point (pH 9.2) via a pH meter, and thus calculating the amino acid nitrogen content by the consuming volume of standard NaOH solution.

### Cultivable microbes counting

The cultivable microbes in *Daqu* were quantified using traditional dilution and plating method, following our previous protocols with minor adjustments (Dong et al., 2022). Specifically, *Bacillus*, lactic acid bacteria (LABs), yeasts, and molds were targeted for detection. Ten grams of *Daqu* powder were combined with 90 mL of sterile H_2_O and shaken at 160 rpm for 30 min. Subsequently, a gradient dilution was performed, and 100 μL of the diluted suspension was plated on respective agar plates to cultivate cultivable microbes. LB agar, YPD agar (with ampicillin), MRS agar (with nystatin), and PDA (with ampicillin) were employed to support the growth of *Bacillus*, LABs, yeasts, and molds, respectively, under appropriate conditions. Following the designated incubation period, the number of visible colonies on the respective plates was enumerated.

### Fermenting vitalities analysis

The fermenting vitalities of *Daqu* was monitored by detecting neutral protease activity, α-amylase activity, glucoamylase activity, and fermentation ability. Thereinto, the neutral protease activity was determined via colorimetry at 680 nm using Folin–Ciocalteu method based on our previous study (Dong et al., 2020). The α-amylase activity was detected based on iodine-starch colorimetric assay, and the decrease in absorbance at 620 nm was indicative of α-amylase activity. Glucoamylase activity was evaluated by determining the glucose produced from starch degradation via DNS (3,5-dinitrosalicylic acid) method. Additionally, fermentation ability was assessed by quantifying the weight of CO_2_ produced during sorghum juice fermentation inoculated with *Daqu* powder.

### Flavor substances analysis

The flavor substances in *Daqu* were detected by a head-space solid-phase microextraction and gas chromatography–mass spectrometry (HS-SPMEGC–MS) according to our previous study (Dong et al., 2022). In this process, 2.0 g of *Daqu* powder and 3.0 g NaCl were combined in 10 mL of 10% ethanol within a headspace bottle, then internal standard substances (2-octanol and 2-ethyl hexanol) were added. After that, the flavor substances in mixture were extracted by an automatic headspace sampling system (Multipurpose Sample MPS 2XL) at 50°C for 45 min. Subsequently, the SPME fiber was introduced into the injection port, set at 250°C, for a duration of 5 min. Compounds separation was achieved using an Agilent HP-5 column (30 m × 0.25 mm; 0.25 μm film thickness) and a DB-FFAP column (60 m × 0.25 mm; 0.25 μm film thickness), followed by analysis via GC–MS analysis employing the Agilent 7890B GC system and the 5977C mass selective detector. Compounds identification was carried out by comparing mass spectral profiles with a match quality of ≥80 in the NIST14 database.

### Microbial community analysis based on amplicon sequencing

The third-generation amplicon sequencing was employed to analyze the composition, structure, and succession of microbial community in three types of *Daqu* during maturation, specifically on day 0, 30, 90, and 180. Initially, total DNA was extracted from 0.2 grams of *Daqu* powder using the E.Z.N.A.^®^ Soil DNA Kit (Omega Bio-Tek, United States) as per the provided instructions. After the concentration and quality assessment of total DNA were conducted, it served as the template for library construction. For the full-length 16S rDNA library of bacteria, amplification was performed using Q5 high-fidelity DNA polymerase (NEB, United States) with primers 27F (AGRGTTTGATYNTGGCTCAG) and 1492R (TASGGHTACCTTGTTASGACTT) (Callahan et al., 2019). The amplification parameters were as follows: 98°C for 5 min, 98°C for 30 s, 55°C for 30 s, and 72°C for 45 s for 30 cycles, and final at 72°C for 5 min. Simultaneously, the full-length ITS library of fungi was constructed by Q5 high-fidelity DNA polymerase using primers ITS1-F (CTTGGTCATTTAGAGGAAGTAA) and ITS4 (TCCTCCGCTTATTGATATGC) (Kuang et al., 2021). The amplification parameters for this library were: 98°C for 5 min, 98°C for 30 s, 53°C for 30 s, and 72°C for 20 s for 30 cycles, with a final step at 72°C for 5 min. Subsequently, the purified PCR products of these two libraries were subjected to high-throughput sequencing (HTS) using the PacBio Sequel II platform. The post-sequencing data were analyzed bioinformatically using QIIME2, with the main analytical workflow referenced from Yang’s study (Yang et al., 2024).

### Data availability and statistical analysis

The raw data generated from HTS has been securely archived in NCBI under the BioProject accession number PRJNA 1034761 and PRJNA 1034762. Statistical analyses in this study involved data processing with Excel (version 2019), and the determination of significance was conducted using Origin (version 9.0), with a significance threshold set at 0.05.

## Results and discussions

### Physicochemical properties

**Figure 2:**
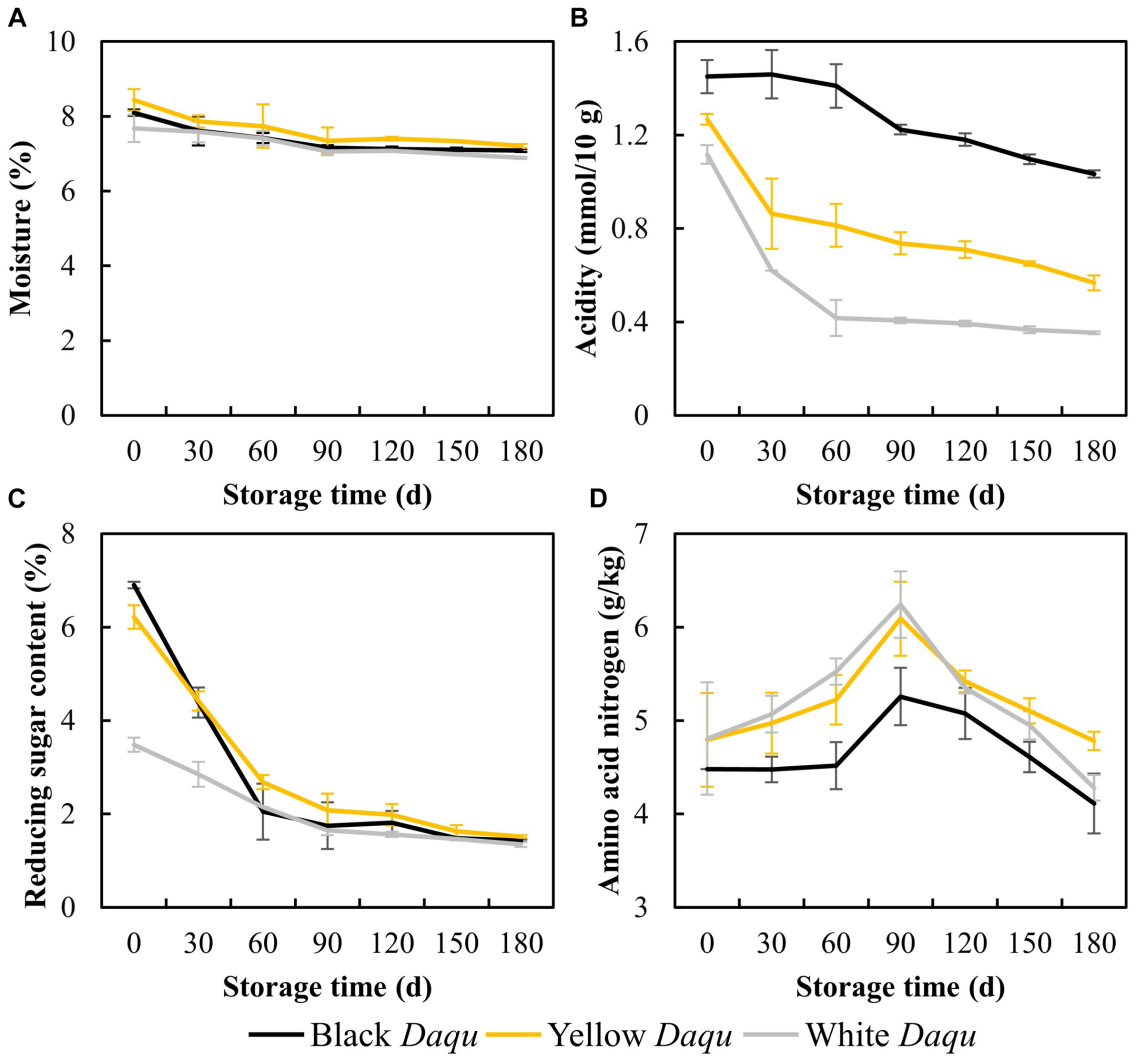
The physicochemical factors of three kinds *Daqu* during maturation, including moisture **(A)**, acidity **(B)**, reducing sugar **(C)**, and amino acid nitrogen **(D)**.

The physicochemical properties of the three types of *Daqu* during the maturation process were shown in Figure 2, emphasizing key parameters such as moisture content, acidity, reducing sugar, and amino acid nitrogen levels. A gradual reduction in moisture content was observed among the three types of *Daqu*, with levels decreasing from 8.09 to 7.08% for black *Daqu*, 8.43 to 7.20% for yellow *Daqu*, and 7.68 to 6.89% for white *Daqu* (Figure 2A). Notably, all three types reached a final moisture content below 9%, adhering to the standard for qualified *Daqu*, preserving enzymatic and microbial activities (Paredes-López et al., 1988; Xia et al., 2022). While the dropping trend of acidity during maturation was similar across the three colored *Daqu*, black *Daqu* exhibited the highest acidity, white *Daqu* the lowest, and yellow *Daqu* intermediate, consistent with findings in Zhang’s study (Zhang et al., 2022). At the end of the maturation period, the acidity values for these three types of *Daqu* were 1.03, 0.56, and 0.35 mmol/10 g, respectively (Figure 2B), which were lower than those in previous studies (Deng et al., 2020; Zhang et al., 2022). In terms of reducing sugar, initial content varied significantly among the *Daqu* types but showcased an overall dropping trend, stabilizing at a low level of approximately 1.40% by the end of maturation (Figure 2C). This low reducing sugar content is conducive to microbial stabilization but insufficient for microbial growth in *Daqu*. Amino acid nitrogen content showed an initial increase followed by a decrease, with black *Daqu* having the lowest final content (4.11 g/kg), white *Daqu* next (4.28 g/kg), and yellow *Daqu* having the highest content (4.78 g/kg) (Figure 2D).

### Cultivable microbes counting

The counts of cultivable microbes in the three-color *Daqu* manifested an overall raising trend followed by dwindling during the maturation process (Table 1), aligning with the previously noted decrease in moisture content (Figure 2A). In cases of *Bacillus*, the number ranged from 6.33 × 10^5^ to 9.03 × 10^8^ cfu/g, with white *Daqu* exhibiting the highest count, followed by yellow *Daqu*, and black *Daqu* (Table 1). This *Bacillus* count falls within a reasonable range reported in previous studies, where the maximal order of magnitudes for *Bacillus* is around 10^8^ in *Daqu* (Sakandar et al., 2020). The prevalence of *Bacillus* in cultivable microbes may be attributed to its ability to survive in harsh conditions (Liu et al., 2019; Sakandar et al., 2020). The number of LABs were lower (6.30 × 10^5^ to 6.21 × 10^7^ cfu/g) than those of *Bacillus* in the three *Daqu* and exhibited an opposite trend, with black *Daqu* having the highest LAB count, followed by yellow *Daqu* and white *Daqu* (Table 1). This finding aligns with previous research indicating that LABs contribute to substrate acidity, inhibiting the growth of *Bacillus* (Zou et al., 2018). The quantity of cultivable fungi was significantly lower than that of bacteria, with yeast counts ranging from 3.62 × 10^2^ to 3.32 × 10^5^ cfu/g and mold counts varying from 7.66 × 10^2^ to 3.53 × 10^6^ cfu/g (Table 1). Among these, white *Daqu* had the highest counts of yeast and mold, while black *Daqu* had the lowest counts, suggesting a synergistic effect of yeast and mold during the maturation process.

**Table 1:** The counts of cultivable microbes in three-color *Daqu* samples during maturation.

Maturation time (d)	*Bacillus* (cfu/g)			LABs (cfu/g)		
	Black *Daqu*	Yellow *Daqu*	White *Daqu*	Black *Daqu*	Yellow *Daqu*	White *Daqu*
0	(3.06 ± 0.20) × 10^7^	(1.49 ± 0.20) × 10^7^	(1.25 ± 0.20) × 10^8^	(6.21 ± 0.10) × 10^7^	(4.31 ± 0.11) × 10^7^	(4.54 ± 0.24) × 10^6^
30	(8.69 ± 0.40) × 10^7^	(8.82 ± 0.40) × 10^7^	(2.43 ± 0.20) × 10^8^	(4.32 ± 0.12) × 10^7^	(3.94 ± 0.40) × 10^6^	(2.72 ± 0.30) × 10^6^
60	(2.58 ± 0.27) × 10^8^	(3.66 ± 0.24) × 10^8^	(4.30 ± 0.15) × 10^8^	(4.05 ± 0.27) × 10^7^	(3.28 ± 0.20) × 10^6^	(2.52 ± 0.20) × 10^6^
90	(4.93 ± 0.52) × 10^8^	(5.72 ± 0.59) × 10^8^	(9.03 ± 0.05) × 10^8^	(3.87 ± 0.10) × 10^7^	(2.87 ± 0.20) × 10^6^	(2.43 ± 0.23) × 10^6^
120	(1.27 ± 0.32) × 10^7^	(3.01 ± 0.21) × 10^7^	(5.76 ± 0.68) × 10^8^	(3.28 ± 0.10) × 10^7^	(2.61 ± 0.50) × 10^6^	(2.07 ± 0.40) × 10^6^
150	(5.50 ± 0.31) × 10^6^	(2.25 ± 0.41) × 10^7^	(4.71 ± 0.46) × 10^8^	(2.87 ± 0.07) × 10^7^	(2.07 ± 0.40) × 10^6^	(1.89 ± 0.30) × 10^6^
180	(6.33 ± 0.25) × 10^5^	(9.02 ± 0.85) × 10^6^	(3.63 ± 0.32) × 10^8^	(2.07 ± 0.10) × 10^7^	(1.80 ± 0.05) × 10^6^	(6.30 ± 0.05) × 10^5^
Maturation time (d)	Yeasts (cfu/g)			Molds (cfu/g)		
	Black *Daqu*	Yellow *Daqu*	White *Daqu*	Black *Daqu*	Yellow *Daqu*	White *Daqu*
0	(1.01 ± 0.01) × 10^3^	(1.08 ± 0.01) × 10^3^	(2.95 ± 0.40) × 10^4^	(3.21 ± 0.11) × 10^6^	(5.91 ± 0.13) × 10^4^	(3.53 ± 0.01) × 10^6^
30	(8.70 ± 0.80) × 10^3^	(9.10 ± 0.90) × 10^3^	(3.32 ± 0.27) × 10^5^	(8.92 ± 0.40) × 10^5^	(5.54 ± 0.19) × 10^4^	(9.70 ± 0.96) × 10^5^
60	(3.60 ± 0.01) × 10^3^	(9.00 ± 0.04) × 10^3^	(2.27 ± 0.20) × 10^5^	(2.64 ± 0.37) × 10^4^	(3.83 ± 0.25) × 10^4^	(8.73 ± 1.57) × 10^5^
90	(2.70 ± 0.01) × 10^3^	(8.70 ± 0.05) × 10^3^	(8.57 ± 0.89) × 10^4^	(9.77 ± 1.11) × 10^3^	(1.78 ± 0.26) × 10^4^	(6.13 ± 0.33) × 10^5^
120	(9.06 ± 1.32) × 10^2^	(9.60 ± 1.00) × 10^2^	(5.40 ± 0.20) × 10^4^	(5.40 ± 0.05) × 10^3^	(1.66 ± 0.16) × 10^4^	(5.70 ± 0.52) × 10^5^
150	(8.10 ± 1.21) × 10^2^	(9.00 ± 1.41) × 10^2^	(1.80 ± 0.20) × 10^4^	(1.60 ± 0.01) × 10^3^	(6.30 ± 0.60) × 10^3^	(2.10 ± 0.42) × 10^5^
180	(3.62 ± 0.80) × 10^2^	(8.10 ± 2.85) × 10^2^	(9.00 ± 1.00) × 10^3^	(7.66 ± 2.20) × 10^2^	(2.70 ± 0.30) × 10^3^	(1.68 ± 0.58) × 10^5^

### Fermenting vitalities

**Figure 3:**
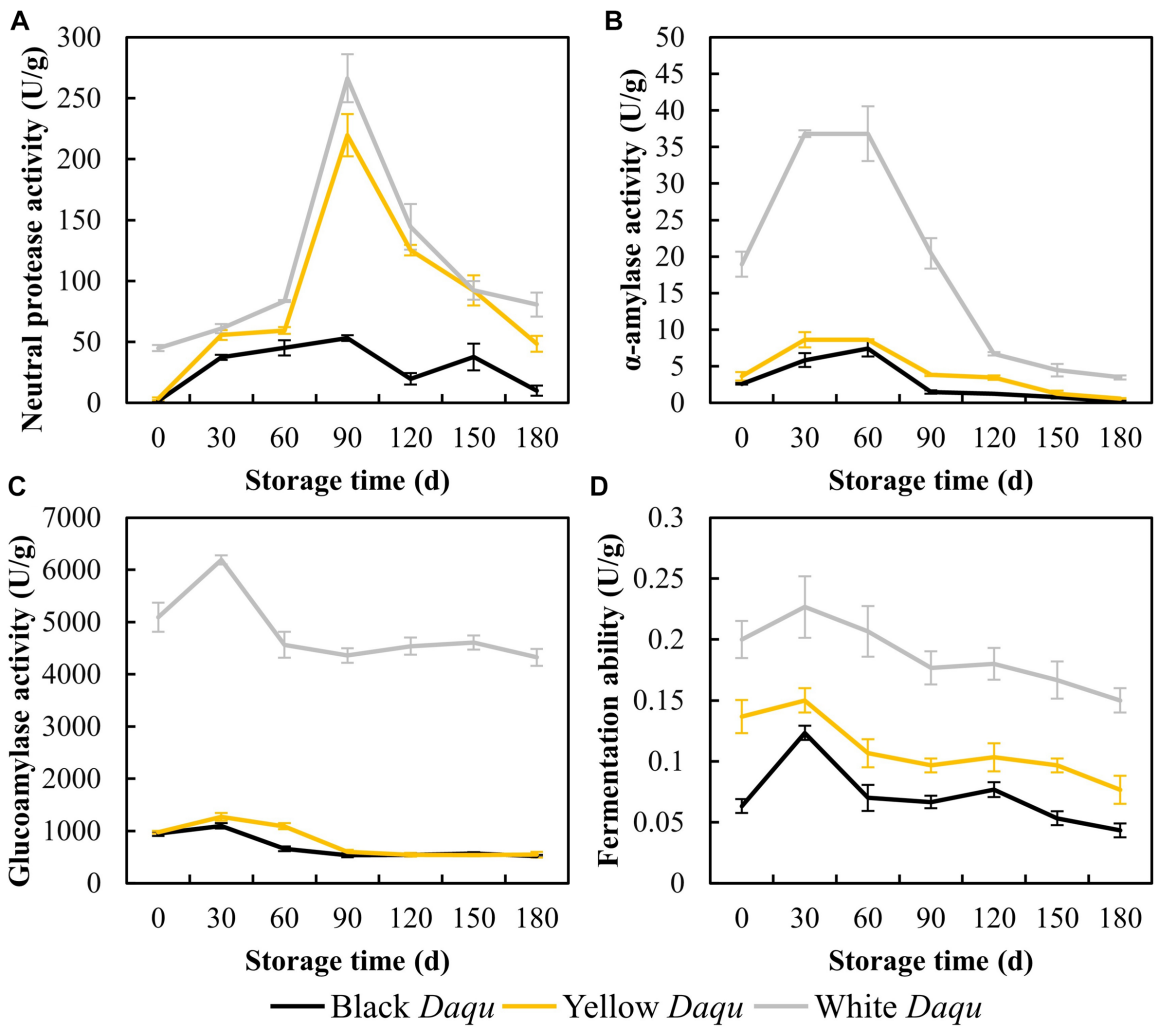
The fermenting vitalities of three kinds *Daqu* during maturation, including neutral protease **(A)**, α-amylase **(B)**, glucoamylase activity **(C)**, and fermenting ability **(D)**.

The fermenting vitalities of *Daqu* play a crucial role in the production of sauce-flavor baijiu, influencing the initiation of baijiu fermentation (Wang B. et al., 2018; Li H. et al., 2023). In this study, we conducted a comprehensive analysis of neutral protease, α-amylase, glucoamylase, and fermentation ability in the three types of *Daqu* to evaluate their fermenting vitalities. Neutral protease activity displayed an initial increase followed by a decrease in all three types of *Daqu* (Figure 3A). White *Daqu* exhibited the final activity at 80.61 U/g, followed by yellow *Daqu* at 48.51 U/g, and black *Daqu* at 9.98 U/g on day 180 of maturation (Figure 3A). This result is consistent with the changes in the counts of *Bacillus* mentioned earlier (Table 1), as *Bacillus* is recognized for its superior protease production (Contesini et al., 2017). The activities of α-amylase, glucoamylase, and fermentation vitalities all exhibited similar trends, with initial increases followed by a decline and subsequent stabilization (Figures 3B–D). Overall, white *Daqu* demonstrated the highest activity, followed by yellow *Daqu*, and black *Daqu* had the lowest (Figures 3B–D). These results align with the counts of yeast and mold in cultivable microbes (Table 1), indicating that a higher count of mold and yeast promotes the production of α-amylase and glucoamylase (Wang X. D. et al., 2018), ultimately resulting in stronger fermentation activity (Yang et al., 2022). In summary, the analysis of the fermenting vitalities of the three types of *Daqu* suggests that white *Daqu* has the highest quality and provides stronger fermentation capabilities, contributing to the initiation of sauce-flavor baijiu brewing.

### Flavor substances

**Figure 4:**
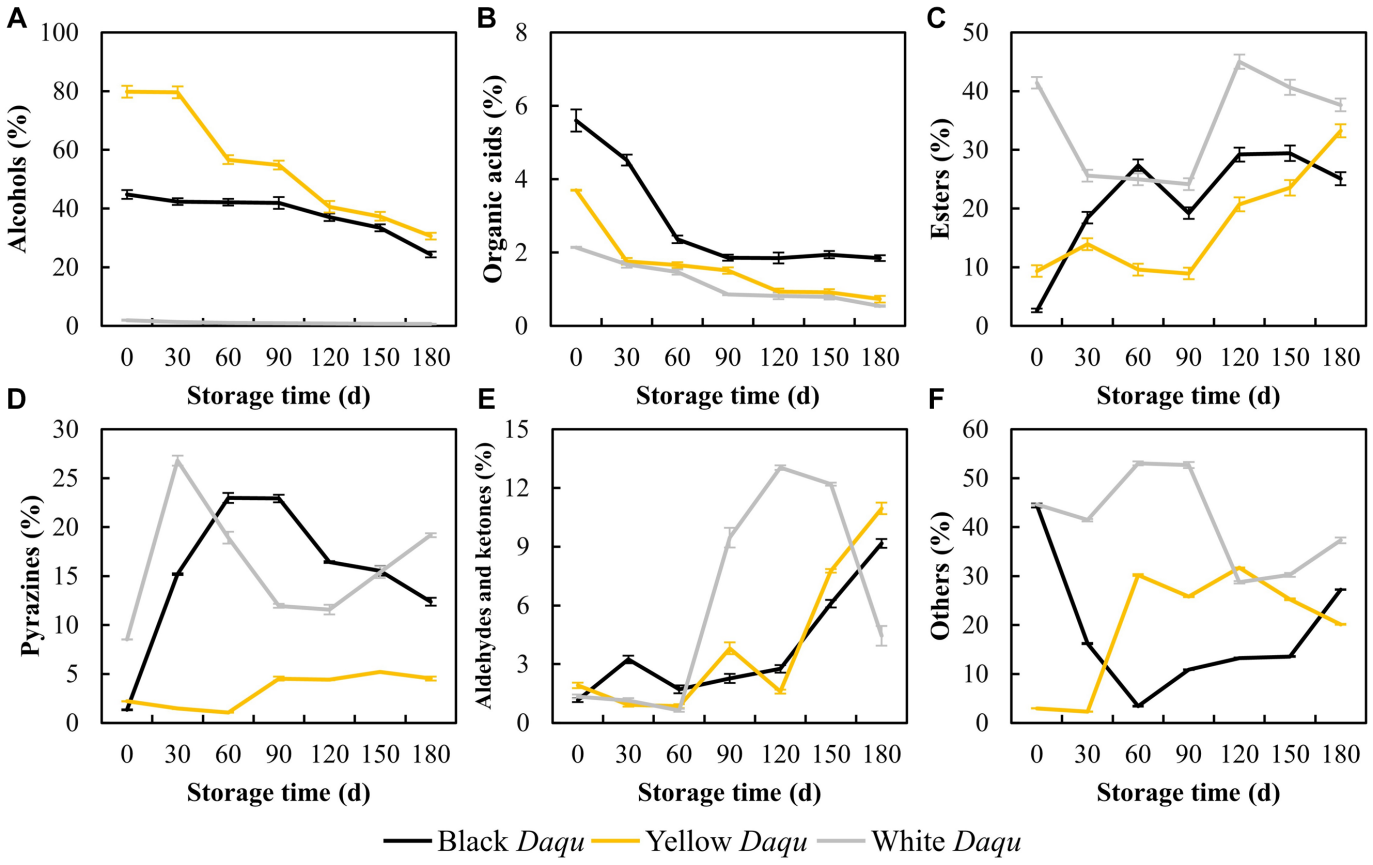
The variation of main flavor compounds in three kinds *Daqu* during maturation. Here, the alcohols **(A)**, acids **(B)**, esters **(C)**, pyrazines **(D)**, aldehydes and ketones **(E)**, and the others **(F)** were presented.

The flavor substances in *Daqu* serve as the precursors to determine the final flavor and characteristics of baijiu (Li H. et al., 2023). A total of 160 different chemicals were detected, and the fluctuations in flavor substances during the maturation of the three types of Daqu are intricate. Here, we focused on analyzing alcohols, organic acids, esters, pyrazines, aldehydes and ketones (Figure 4). The content of alcohols in the colored *Daqu* all exhibited an overall decreasing trend throughout the maturation process (Figure 4A). Yellow *Daqu* initially had the highest alcohols content (79.79%) on day 0, dropping to 30.36% on day 180, while black *Daqu* started with 44.82%, decreasing to 24.33% at maturity (Figure 4A). In contrast, white *Daqu* showed the lowest initial alcohols content of 1.97%, declining to 0.68% on day 180 (Figure 4A). Organic acids content also decreased during maturation in these three types of *Daqu*, albeit with a milder decline than the variation observed in alcohols, ranging from 1.60 to 3.75% (Figure 4B). Ultimately, black *Daqu* had the highest organic acids content (1.85%), followed by yellow *Daqu* (0.73%), and white *Daqu* (0.54%) at the end of maturation (Figure 4B), in line with changes in acidity mentioned earlier (Figure 2B). Esters content varied significantly among the three types of *Daqu*, with white *Daqu* having the highest overall content, fluctuating throughout maturation, while black *Daqu* and yellow *Daqu* showed a similar upward trend (Figure 4C). Thus, the decline in alcohols and organic acids observed here can be attributed to their substantial consumption in the formation of esters. Pyrazine substances, crucial flavor compounds in sauce-flavor baijiu, contribute to pleasant aromas of roasted nuts and cocoa (Mortzfeld et al., 2020; Shi X. et al., 2022). They were considered one type of the characteristic flavor components that differentiate sauce-flavor baijiu from other types of baijiu (Zhang et al., 2013; Niu et al., 2022). The content of pyrazines in the three-color *Daqu* showed big differences (Figure 4D). White *Daqu* and black *Daqu* maintained high levels of pyrazines (>10%), with white *Daqu* reaching 19.18% and black *Daqu* at 12.37% on day 180 (Figure 4D). In contrast, the content of pyrazines in yellow *Daqu* remained at low level, reaching 4.53% on day 180 (Figure 4D). Aldehydes and ketones exhibited an increasing trend in both black *Daqu* and yellow *Daqu*, reaching 9.17 and 10.96% at the end of maturation, respectively, whereas white *Daqu* experienced a sharp increase to 13.04% during day 60–120, followed by a rapid decrease to 4.45% in the end (Figure 4E). Other flavor substances exhibited varying changes among the three types of *Daqu* (Figure 4F). In summary, the diverse changes in flavor substances during the maturation process of the three types of *Daqu* indicate each type’s unique characteristics, suggesting potential combinations based on differences in flavor substances for sauce-flavor baijiu brewing.

### Difference in microbial community

Through third-generation HTS, we analyzed the microbial community of the three-color *Daqu* during maturation. The results indicated that the sequencing coverage exceeded 0.991 (Table 2), demonstrating the depth and reliability of the amplicon sequencing. Regarding bacterial α diversity, the Chao1 and Shannon indices for the three types of *Daqu* generally exhibited a trend of initial decrease followed by an increase as they mature (Table 2). Overall, the Chao1 richness was highest in black *Daqu*, followed by white *Daqu*, and lowest in yellow *Daqu*. The Shannon index was higher in black and white *Daqu* compared to yellow Daqu. For fungal α diversity, the Chao1 richness decreased for all three types of *Daqu*, indicating a decline in fungal richness during *Daqu* maturation. This decrease may be associated with the decline in moisture content during *Daqu* maturation since the lower moisture levels, below 9%, can reduce free water in microbial cells, hampering their growth and reproduction. In contrast, the fungal Shannon index remained relatively stable, with white *Daqu* exhibiting the highest value, suggesting a more even distribution of fungi during maturation. In summary, the primary diversity differences among the three types of *Daqu* are reflected in the Chao1 richness, with less variation in the Shannon index (Table 2).

**Table 2:** The α diversity of three-color *Daqu* samples during maturation.

ID	Bacteria			Fungi		
Samples	Chao1	Shannon	Good coverage	Chao1	Shannon	Good coverage
Black *Daqu* 0d	371.84 ± 62.77	3.99 ± 0.45	0.991 ± 2.2E-03	360.46 ± 43.67	1.28 ± 0.19	0.992 ± 2.3E-03
Black *Daqu* 30d	342.08 ± 35.92	2.92 ± 0.41	0.999 ± 1.6E-03	278.06 ± 80.61	1.92 ± 0.48	0.995 ± 5.7E-03
Black *Daqu* 90d	290.96 ± 60.39	2.92 ± 0.33	0.992 ± 6.3E-03	257.13 ± 90.32	1.01 ± 0.36	0.994 ± 4.7E-03
Black *Daqu* 180d	430.04 ± 78.09	3.46 ± 0.79	0.991 ± 5.6E-04	95.61 ± 20.25	2.50 ± 0.36	0.999 ± 4.2E-04
Yellow *Daqu* 0d	150.86 ± 28.83	1.94 ± 0.44	0.997 ± 5.8E-04	466.74 ± 113.64	3.76 ± 0.86	0.991 ± 3.9E-03
Yellow *Daqu* 30d	132.89 ± 29.12	1.47 ± 0.29	0.997 ± 3.0E-03	259.80 ± 75.99	1.82 ± 0.22	0.995 ± 5.6E-03
Yellow *Daqu* 90d	106.87 ± 35.98	2.06 ± 0.42	0.998 ± 8.0E-04	190.64 ± 72.76	1.49 ± 0.42	0.995 ± 4.5E-03
Yellow *Daqu* 180d	357.77 ± 82.34	3.08 ± 0.57	0.993 ± 1.2E-03	61.12 ± 16.99	2.54 ± 0.28	0.999 ± 4.4E-04
White *Daqu* 0d	245.37 ± 61.34	3.23 ± 0.29	0.996 ± 1.1E-03	350.42 ± 20.23	2.08 ± 0.34	0.997 ± 1.7E-03
White *Daqu* 30d	243.42 ± 34.99	3.96 ± 0.33	0.995 ± 2.9E-04	310.92 ± 56.51	4.14 ± 0.65	0.995 ± 2.0E-03
White *Daqu* 90d	241.58 ± 50.66	2.98 ± 0.22	0.993 ± 2.9E-03	206.15 ± 36.84	2.21 ± 0.59	0.994 ± 5.2E-03
White *Daqu* 180d	280.74 ± 80.69	3.55 ± 0.35	0.994 ± 1.4E-03	101.48 ± 33.71	2.79 ± 0.81	0.998 ± 9.7E-04

To further assess the structure of microbial community during *Daqu* maturation, a Principal Component Analysis (PCA) based on Bray-Curtis distances was conducted. The β diversity results revealed that the structural differences in bacterial community among the three types of *Daqu* were more pronounced than for fungal community. In the bacterial β diversity plot, samples representing the three types of *Daqu* were more scattered, indicating greater differences (Supplementary Figure S1A). In contrast, in the fungal β diversity plot, samples from all three *Daqu* types clustered closely and were not easily distinguishable (Supplementary Figure S1B). This implies that the differences in microbial diversity mainly exist in the bacterial community rather than the fungal community.

**Figure 5:**
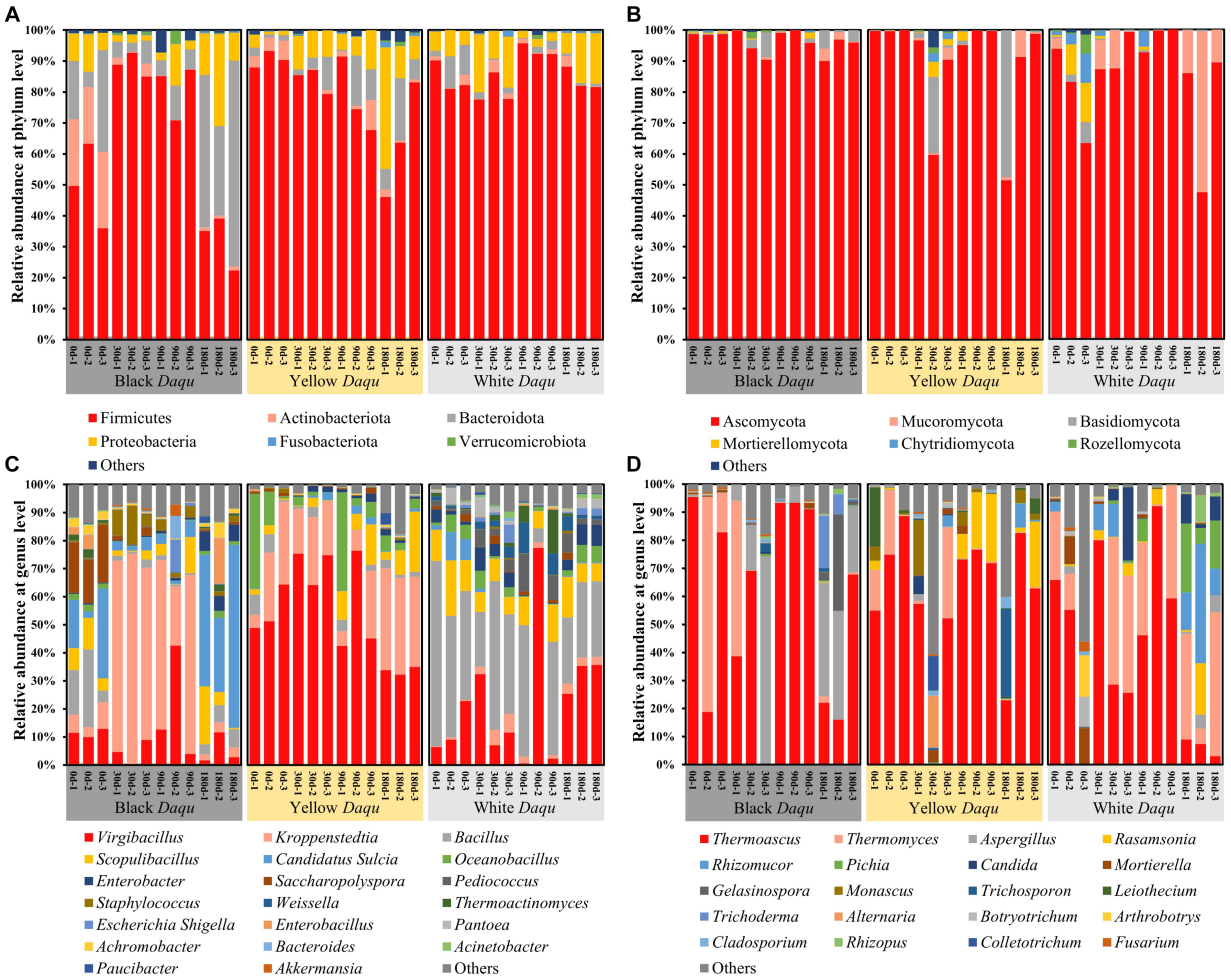
The microbial community of three kinds of *Daqu* during maturation, including bacterial phylum level **(A)**, fungal phylum level **(B)**, bacterial genus level **(C)**, and fungal genus level **(D)**.

To investigate the composition of microbial community in *Daqu*, differences in taxa at the phylum and genus levels were analyzed during the maturation of the three-color *Daqu*. For bacterial composition, Firmicutes, Actinobacteriota, Bacteroidota, Proteobacteria, Fusobacteriota, and Verrucomicrobiota constituted the predominant phyla (Figure 5A). Among them, Firmicutes emerged as the most abundant phylum, which was congruent with Gan’s study (Gan et al., 2019). However, the abundance of Firmicutes varied among three kinds of *Daqu*. In black *Daqu*, there was an initial increase followed by decrease (49.59 to 88.80 to 32.17%) (Figure 5A). Yellow *Daqu* exhibited a minor decline from 97.61 to 91.07%, while Firmicutes remained relatively stable in white Daqu, with abundances consistently above 80.52% (Figure 5A). As for fungal composition, the predominant phyla included Ascomycota, Mucoromycota, Basidiomycota, Mortierellomycota, Chytridiomycota, and Rozellomycota (Figure 5B). Ascomycota was the most dominant phylum, collaborating with previous studies (Deng et al., 2020; Zhang et al., 2022). The abundance of Ascomycota in black *Daqu* maintained above 94.29%, yellow *Daqu* exhibited fluctuations ranging from 80.51 to 99.77%, and white *Daqu* displayed an initial increase followed by a decrease (80.03 to 97.43 to 74.23%) (Figure 5B).

At the genus level, the composition of microbial community in the three-color *Daqu* showed even greater differences (Figures 5C,D). For bacterial, *Virgibacillus*, *Kroppenstedtia*, *Bacillus*, *Scopulibacillus*, *Candidatus Sulcia*, *Oceanobacillus*, *Enterobacter*, *Saccharopolyspora*, *Pediococcus*, and *Staphylococcus* were the top 10 abundant genera (Figure 5C). However, their relative abundances in the microbial community changed significantly during *Daqu* maturation. In black *Daqu*, *Kroppenstedtia* (31.58%), *Candidatus Sulcia* (17.49%), and *Virgibacillus* (10.27%) constituted the top 3 dominant bacterial genera (Figure 5C). *Kroppenstedtia*, in particular, was the most dominated genera in black *Daqu*, and its relative abundance rapidly increased from 6.48% (day 0) to 68.20% (day 30), followed by a continue decrease to 3.11% on day 180 (Figure 5C). Previous studies have unveiled that *Kroppenstedtia* is the core microbe in connection with the biosynthesis of organic acids (lactic acid and short chain fatty acids) (Zhu C. et al., 2023; Zhang et al., 2024), explaining the highest acidity observed in black Daqu during maturation. In yellow *Daqu*, *Virgibacillus* (54.66%), *Kroppenstedtia* (21.47%), and *Oceanobacillus* (8.57%) made up the top 3 dominant bacterial genera (Figure 5C). *Virgibacillus* was the main dominant genus in yellow *Daqu*, with its relative abundance quickly rosing from 54.83% (day 0) to 71.45% (day 30) and then gradually dropping to 33.67% (day 180) (Figure 5C). This result differs from previous studies where *Virgibacillus* was a dominant bacterium but not the most dominant in *Daqu* (Zhu C. et al., 2023; Zhu Q. et al., 2023). The roles and functions of *Virgibacillus* in *Daqu* production and baijiu brewing are not clear and needs further study. In white *Daqu*, *Bacillus* (35.53%), *Virgibacillus* (22.13%), and *Scopulibacillus* (10.01%) were the top three dominant bacterial genera (Figure 5C). *Bacillus* was the most dominant genus, and its relative abundance gradually decreased during maturation (from 49.44 to 25.86%) (Figure 5C). Despite the decreasing trend, the abundance of *Bacillus* remained above 25%, suggesting that it could still function effectively. *Bacillus* is known for secreting amylase and proteases and playing essential roles in liquefaction and saccharification (Ding et al., 2013; Wang et al., 2014, 2020), contributing to the higher fermenting vitalities observed in white *Daqu* (Figure 3). In addition, the average abundance of *Kroppenstedtia* in three kinds of *Daqu* followed the order: black *Daqu*, yellow *Daqu*, and white *Daqu*. *Kroppenstedtia* was found to be positively correlated with the production of various amino acids in *Daqu* (Zhu C. et al., 2023), which might promote the Maillard reaction with reducing sugar, leading to the formation of the yellow-brown color and contributed to the color difference in these three-color *Daqu*. Therefore, *Kroppenstedtia*, *Virgibacillus*, and *Bacillus* were identified as the three core bacteria in these colored *Daqu*, with *Kroppenstedtia* being the potential key microbe related to color formation.

For fungal genera, differences were smaller than those of bacteria among the three types of *Daqu*. The top 10 abundant fungal genera included *Thermoascus*, *Thermomyces*, *Aspergillus*, *Rasamsonia*, *Rhizomucor*, *Pichia*, *Candida*, *Mortierella*, *Gelasinospora*, and *Monascus* (Figure 5D). Among them, *Thermoascus* was the most dominant genera shared in all three types of *Daqu*, which was similar to Zhu’s study (Zhu C. et al., 2022). The relative abundance of *Thermoascus* changed in the same manner, initially decreasing, then increasing, and finally decreasing again (Figure 5D). In black *Daqu*, *Thermoascus* (57.38%), *Aspergillus* (17.18%), and *Thermomyces* (12.48%) constituted the top 3 dominant fungal genera, and the relative abundance of *Thermoascus* ranged from 35.92 to 92.58% (Figure 5D). In yellow *Daqu*, the top 3 dominant fungal genera belonged to *Thermoascus* (59.84%), *Rasamsonia* (6.85%), and *Thermomyces* (6.08%), whereas the abundance of *Thermoascus* varied from 36.51 to 73.91% (Figure 5D). In white *Daqu*, *Thermoascus* (39.35%), *Thermomyces* (25.01%), and *Rhizomucor* (7.50%) comprised the top 3 fungal genera, while the abundance of *Thermoascus* fluctuated from 6.44 to 65.88% (Figure 5D). Here, *Thermoascus* is known to produce various enzymes such as catalase, endoglucanase, glucosidase, keratinase, and chitinase, which are able to degrade starch, cellulose or protein from raw materials, providing basic nutrition for other microbes’ metabolism that contributed to flavor formation (Jain et al., 2014; Cai et al., 2021). Thus, *Thermoascus* was identified as the most important fungal genera during *Daqu* maturation.

### CCA between microbial community and physicochemical factors

**Figure 6:**
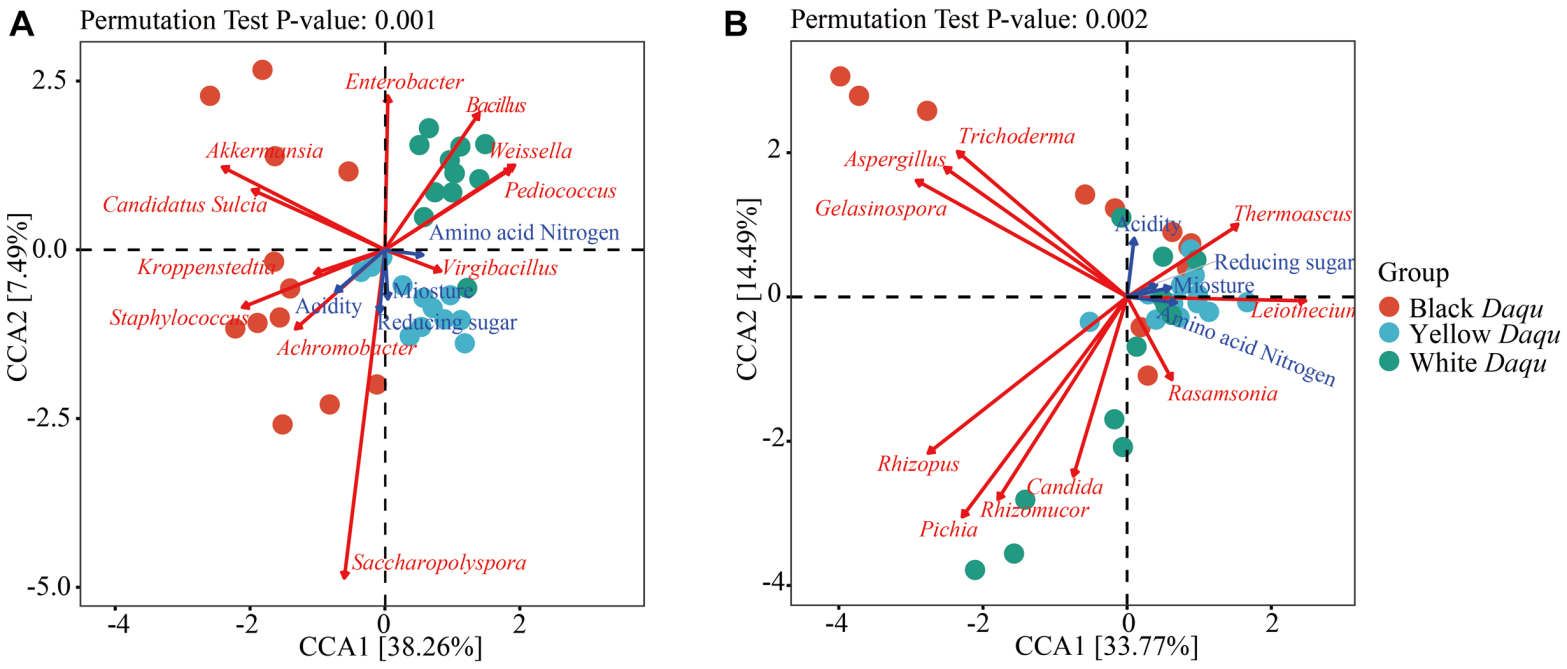
Canonical correlation analysis between microbial community and physicochemical parameters based on the top 20 dominant microbes at genus levels. The different color of dots represented different groups of samples. The blue arrows expressed physicochemical factors, and red arrows stood for core microbes. The length of arrows determines the degree of importance. The correlations of bacterial community **(A)** and fungal community **(B)** to physicochemical parameters.

Physicochemical factors play a crucial role in shaping and influencing the composition and succession of microbial community during spontaneously fermentation processes (Guan et al., 2020). Therefore, canonical correlation analysis (CCA) was employed to examine the impact of physicochemical factors on the microbial community of the three types of *Daqu*. The results, as shown in Figure 6, indicated that acidity had the most substantial impact on microbial community, as the arrow length representing acidity was the longest among the four physicochemical factors. Regarding bacterial communities, the influence of the four physicochemical factors was as follows: acidity > reducing sugar > moisture > amino acid nitrogen (Figure 6A). Specifically, the dominant bacterium *Kroppenstedia* in black *Daqu* showed a positive correlation with acidity, reducing sugar, and moisture, while it had a negative correlation with amino acid nitrogen (Figure 6A). *Kroppenstedtia* is known to correlate with the biosynthesis of organic acids, evidencing its positive relationship with acidity (Zhang et al., 2024). Its negative correlation with amino acid nitrogen contradicted a previous study that found *Kroppenstedtia* to be positively connected to the production of various amino acids in *Daqu* (Zhu C. et al., 2023). This discrepancy might be caused by the color formation in *Daqu*, where amino acids were consumed via the Maillard reaction to form the dark color. In yellow *Daqu*, the dominant bacterium *Virgibacillus* displayed a positive correlation with reducing sugar, moisture, and amino acid nitrogen, but a negative correlation with acidity (Figure 6A). In white *Daqu*, the dominant bacterium *Bacillus* exhibited a positive correlation with amino acid nitrogen but a negative correlation with the other three physicochemical properties (Figure 6A). Here, *Bacillus* is the typical microbe for producing protease (Wang et al., 2014; Contesini et al., 2017), thus hydrolyzing protein to amino acids or small peptides, interpretating this positive correlation. When considering fungal communities, the influence of these four physicochemical factors was as follows: acidity > amino acid nitrogen > moisture > reducing sugar (Figure 6B). The dominant fungus *Thermoascus* in all three types of *Daqu* shared a positive correlation with all four physicochemical factors (Figure 6B), indicating *Thermoascus*’ ability to adapt to the maturation process of *Daqu*. Therefore, acidity was the most critical factor affecting the microbial community of these three kinds of *Daqu* during maturation.

### Relationships between microbial community and flavor substances and fermenting vitalities

**Figure 7:**
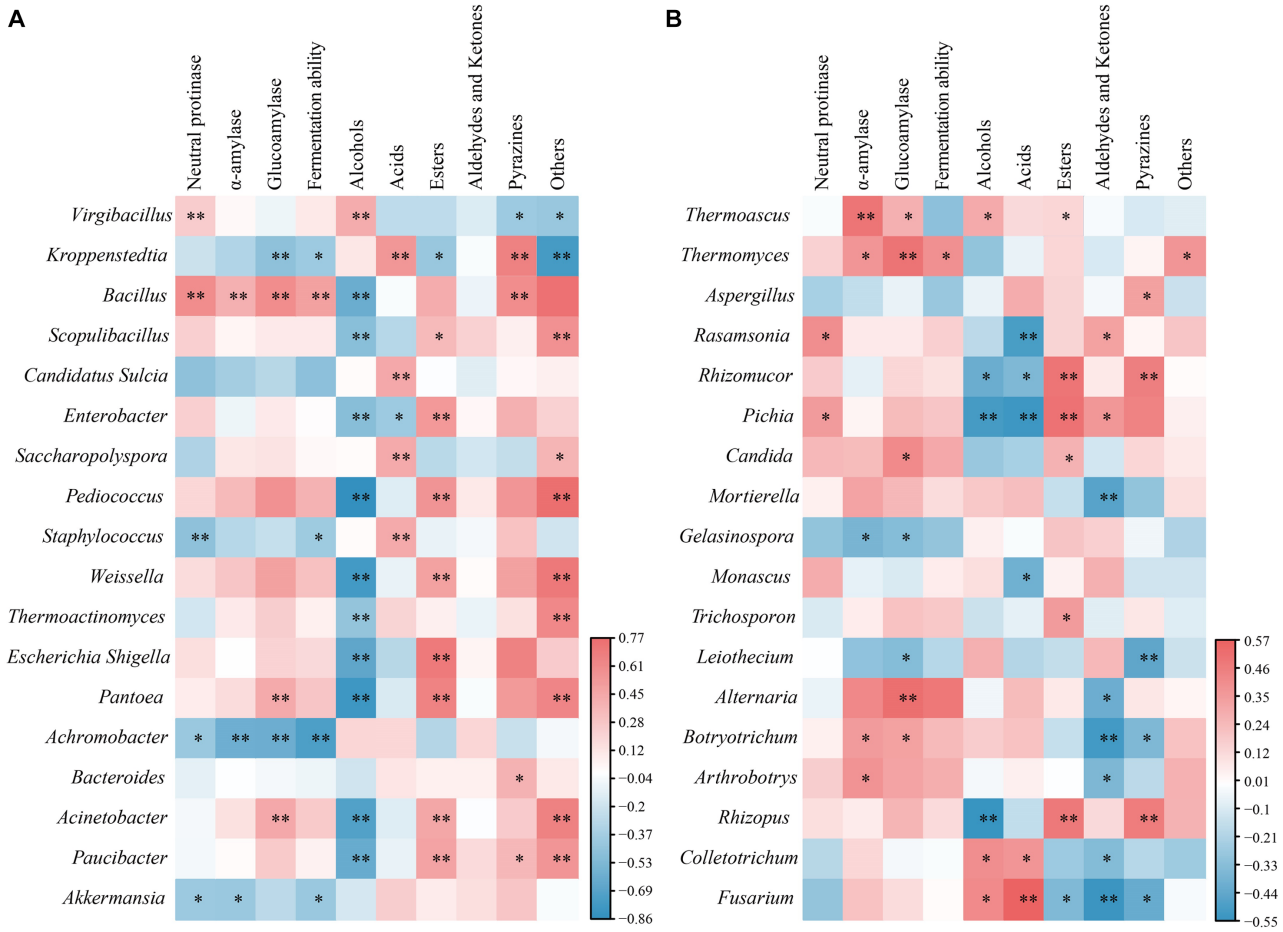
The heatmap of relationships between flavor compounds & activity and dominant microbes. Here, their relationships to bacteria were presented as in **(A)** part, and their relationships to fungi were presented as in **(B)** part. The dominant microbes at genus level (top 20) were selected to analyze the correlations to flavor compounds using Spearman correlation coefficient (*p < 0.05 and **p < 0.01). R value was set at 0.1.

During the maturation of *Daqu*, microbes utilize substrates such as starch, protein, and others, producing a plethora of flavor compounds and various enzymes. These, in turn, have a profound impact on the subsequent production of baijiu, making microbes pivotal in the maturation of *Daqu* and significantly influencing the quality of the final product. To explore these connections, we employed Spearman correlation analysis to unravel the relationships between dominant microbes and flavor compounds, as well as fermenting vitalities. The results, presented in Figure 7, delineated the influence of dominant bacteria and fungi on flavor compounds and fermenting vitalities. In black *Daqu*, the dominant bacterium *Kroppenstedia* exhibited a substantial positive correlation with acids and pyrazines, along with a significant negative correlation with amylase, fermenting vitality, and esters (Figure 7A). These correlations may be attributed to the unique characteristics of *Kroppenstedia*, as reported previously. *Kroppenstedtia* was identified as a core microbe related to the production of organic acids (lactic acid and short chain fatty acids) (Zhu C. et al., 2023; Zhang et al., 2024), and positively correlated with the formation of certain pyrazines such as tetramethylpyrazine and 2,3,5-trimethyl pyrazine (Zhang et al., 2021). Within yellow *Daqu*, the dominant bacterium *Virgibacillus* demonstrated a positive correlation with neutral protease and alcohols, while displaying a negative correlation with pyrazines (Figure 7A). However, the functions of *Virgibacillus* in fermented food is not clear and warrant further investigation. In white *Daqu*, the dominant bacterium *Bacillus* displayed a positive correlation with all enzymes, fermenting vitality, and pyrazines, while holding a negative correlation with alcohols (Figure 7A). *Bacillus* is known to produce various hydrolytic enzymes, including amylase, protease, and lipase, to degrade macromolecules and hence promoting the formation of flavor compounds during baijiu brewing (Jiang et al., 2021; Li Z. et al., 2023). Especially, the pyrazine compounds, providing sauce-flavor baijiu with its unique flavor characteristic, were closely linked to *Bacillus* (He et al., 2019). Considering fungi, the dominant fungus *Thermoascus* across all three types of *Daqu* showed a positive correlation with amylase, glucoamylase, alcohols, and esters (Figure 7B), hinting that *Thermoascus* could promote the formation of flavor by producing various enzymes and collaborating with others functional microbes (Jain et al., 2014; Cai et al., 2021). Notably, the formation of pyrazine substances, characteristic of sauce-flavor baijiu, exhibited a significant positive correlation with *Aspergillus*, *Rhizomucor* and *Rhizopus* (Figure 7B), implying that these fungi also played a crucial role in the formation of pyrazines. This conclusion complements the prevailing belief that *Bacillus* is the core microbe responsible for pyrazine formation, indicating the substantial contributions of molds to this process.

## Conclusion

In this study, the physicochemical properties exhibited similar trends during the maturation of the three types of Daqu, except for acidity, which showed significant differences. A comprehensive analysis of the fermenting capabilities revealed that white *Daqu* attained the highest quality, followed by yellow *Daqu*, while black *Daqu* exhibited the lowest quality. Moreover, the differences in microbial community during maturation were more pronounced for bacteria than for fungi based on the third-generation HTS. *Kroppenstedia*, *Virgibacillus*, and *Bacillus* were the most dominant bacteria in black, yellow, and white *Daqu*, respectively, whereas their dominant fungi all belonged to the *Thermoascus*. Acidity acted as the most notable factor influencing the microbial community. *Kroppenstedtia* was the potential core bacterium affecting the color formation in *Daqu* via Maillard reaction. Furthermore, molds played a pivotal role in pyrazine compounds formation. Therefore, this study provides some novel explanations of color and pyrazine formation from a microbial perspective, laying a foundation for the mechanized *Daqu* production and quality control in the future.

## Data availability statement

The datasets presented in this study can be found in online repositories. The names of the repository/repositories and accession number(s) can be found below: https://www.ncbi.nlm.nih.gov/, PRJNA 1034761 https://www.ncbi.nlm.nih.gov/, PRJNA 1034762.

## Author contributions

WD: Conceptualization, Data curation, Formal analysis, Funding acquisition, Writing – original draft. XY: Data curation, Formal analysis, Writing – review & editing. LW: Methodology, Software, Writing – review & editing. MZ: Investigation, Methodology, Writing – review & editing. JM: Data curation, Software, Writing – review & editing. JL: Conceptualization, Data curation, Writing – review & editing. YF: Investigation, Resources, Writing – review & editing. SZ: Conceptualization, Resources, Writing – review & editing. QY: Resources, Supervision, Writing – review & editing. YH: Funding acquisition, Supervision, Writing – review & editing. SC: Conceptualization, Funding acquisition, Supervision, Writing – review & editing.
