# Supplementary material for: Unveiling the microbiota of sauce-flavor Daqu and its relationships with flavors and color during maturation
Source: Front Microbiol. 2024 Jan 24;15:1345772. doi: 10.3389/fmicb.2024.1345772 (PMC10848165; doi:10.3389/fmicb.2024.1345772)
Supplement: Supplementary file 1 [file Data_Sheet_1.docx]

***Frontiers in microbiology***

**Supplementary material for**

**Unveiling the microbiota of Sauce-flavor *Daqu* and its relationships with flavors and color during maturation**

Weiwei Dong^1, 2†^, Xiang Yu^1†^, Luyao Wang^1^, Menglin Zou^1^, Jiyuan Ma^1^, Jun Liu^1^, Yanli Feng^1^, Shumiao Zhao^3^, Qiang Yang^2^, Yuanliang Hu^1^*, Shenxi Chen^2^*

^1^ Hubei Key Laboratory of Edible Wild Plants Conservation and Utilization, College of Life Sciences, Hubei Normal University, Huangshi 435002, China.

^2^ Hubei key Laboratory of Quality and Safety of Traditional Chinese Medicine Health Food, Jing Brand Co., Ltd., Daye, Hubei 435100, China.

^3^ State Key Laboratory of Agricultural Microbiology and College of Life Science and Technology, Huazhong Agricultural University, Wuhan 430070, China.

***Corresponding authors:**

Yuanliang Hu, E-mail address: ylhu@hbnu.edu.cn

Shenxi Chen, E-mail address: chenshenxi2006@163.com

^†^ These authors contributed equally to this work


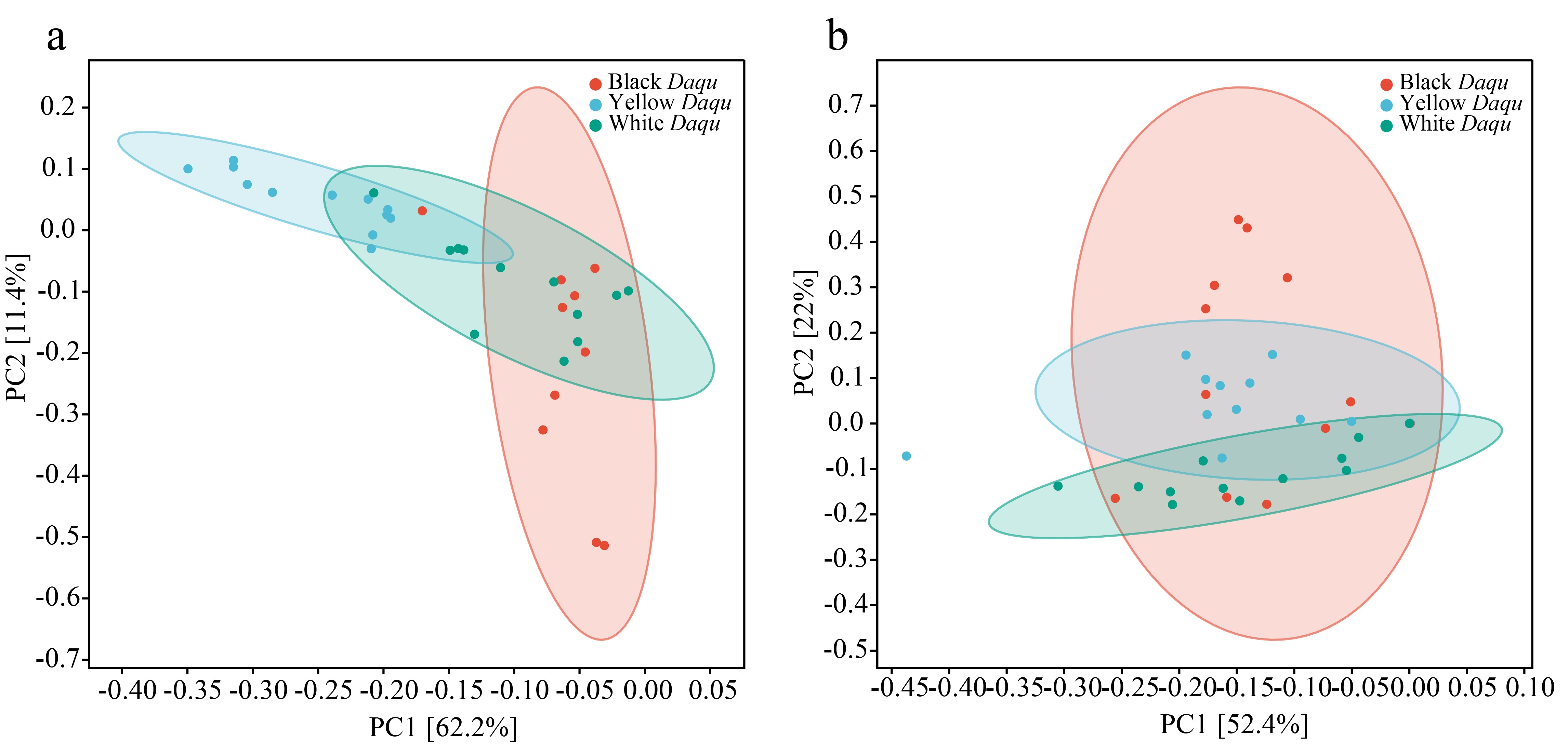


Fig. S1 The microbial community diversity in three kinds of *Daqu* during maturation, bacterial community β-diversity (a), and fungal community β-diversity (b). The Bray-Curtis distance (R2=0.8694. p=0.0001) was used in β-diversity analysis with confidence at 0.95.
